# EUGHS NEWS

**DOI:** 10.7189/jogh.05.020204

**Published:** 2015-12

## KIRSTIE–ANN McPHERSON: MY PERSONAL EXPERIENCE AS A WHO INTERN

For my internship, I spent 6 weeks living in Geneva, working in the Child and Maternal Health department at the World Health Organization (WHO) headquarters. I found the experience to be hugely enjoyable, and a great learning experience in both an academic and personal sense.

Arriving at THE WHO was a daunting experience as this was the first time I was exposed to such a large international organisation. For a young person starting out in the global health world, entering a building where so many world–leading experts work is exciting and scary in equal measures. However, I soon settled with the help of my supervisor. I found the environment to be welcoming and relaxed, and I was able to get to know many of the people working around me. Every person I asked found time to explain their interests and current projects to me, including the director of the nutrition for health and development department who I had a discussion with about governance of the global food industry, an area of personal interest.

As well as gaining an insight into the breadth of work of various experts at THE WHO, I was able to develop a host of my own skills by undertaking my own work and assisting others. My project was focused on evaluating the effectiveness of the CHNRI heath research prioritisation methodology. This systematic method was developed from 2005–2008 and aims to determine global health research priorities in a fairer and more transparent manner with a view to filling the knowledge gaps that result in child mortality remaining high. It features crowd sourcing of expert opinion to establish consensus over research questions of high priority. The main work I did on this topic built on that of a previous intern from the University of Edinburgh. It was based on searches of academic literature relating to the priorities identified in CHNRI exercises about 5 different child health topics: neonatal infection, low birthweight/prematurity, childhood diarrhoea, childhood pneumonia and intrapartum–related neonatal death. We began to determine the interest in these topics in the 3 years’ previous to the publication of these CHNRI exercises, using the number of relevant papers published as a quantitative measure. The comparison value was the numbers published in the years post publication and 3 years of lag time to allow time for studies inspired by the published priorities to be conducted. This was an interesting project, which developed my academic searching skills as well as my critical thinking in relation to developing a method to measure and evaluate an intangible concept like the dissemination of ideas.

**Figure:**
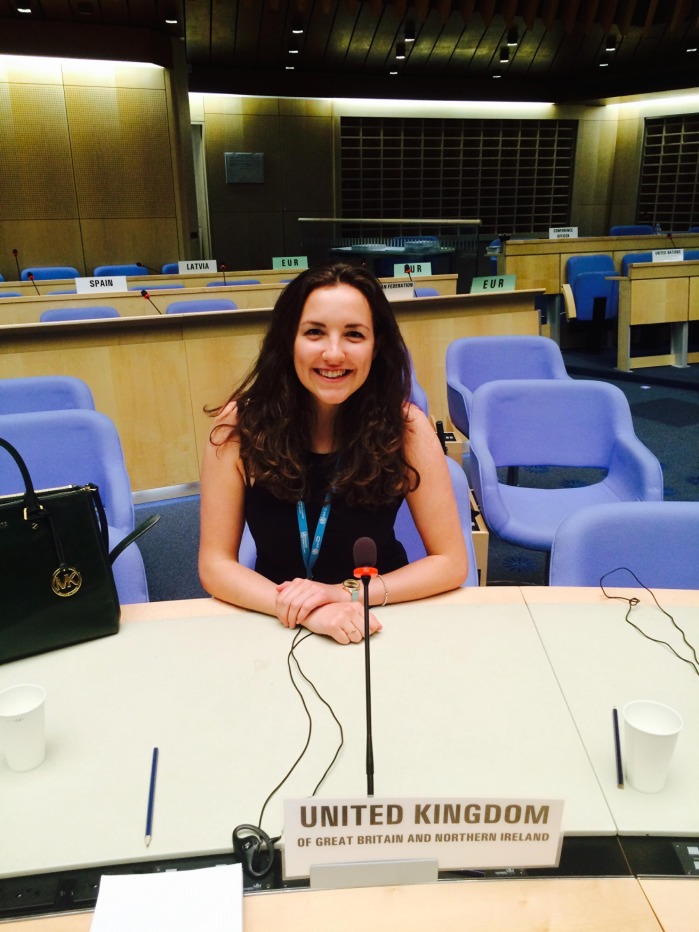
Photo: Kirstie–Ann McPherson at the World Health Organisation

I also spent time in the department facilitating the AMANHI study looking at the global causes of maternal and newborn deaths. Taking part in some of the groundwork of the study accrediting local physicians to perform verbal autopsy highlighted to me the scale of the work involved in performing the research that goes on at THE WHO. These projects take years to design and perform, and spending hours working through a small part of this put into perspective the skill and patience of the people working at THE WHO gathering and sorting complex data from across the world. For me, this further highlighted the importance of THE WHO as a centre point in the field of global health, in this case coordinating in–country field work with that of experts across the world.

My time in Geneva was not only filled with work however, and the intern community at THE WHO was part of making my experience so valuable. There are 300 interns at peak time in summer (none of whom are paid) meaning there is a near constant stream of talks and social events to distract you from working! Spending summer in the heart of Europe was also fantastic as it gave us the opportunity to travel and make the most of every weekend, taking in Lake Annecy, Yvoire, Sciez, Cannes, Lucern and Bern in my time there. However the highlight of my free time was definitely paragliding from the top of Mont Salčve and seeing the beautiful of Geneva from the sky as the sun set.

Overall, I had an inspiring time being submerged in the world of global health for a few short weeks and I am more than grateful to the EUGHS for helping me get the opportunity to experience work and life THE WHO HQ.

## KENNETH McLEAN: MY PERSONAL EXPERIENCE AS A WHO INTERN

I returned to my 3rd year of undergraduate medicine following the completion of an intercalated degree in Epidemiology at the University of Edinburgh. I was enthused to continue to explore my newfound interest in global health and build upon the knowledge and skills I had gained as part of the course. This culminated in the incredible opportunity this summer to undertake a six week internship in the Public Health, Innovation, and Intellectual Property (PHI) Unit of the World Health Organization, Geneva. While there, I was supervised by Erin Sparrow, an experienced technical officer working on influenza virus vaccination, who immediately helped me to feel a welcome and valuable member of the team.

The main focus of my internship was to construct and deliver a survey of vaccine manufacturers to establish the current global influenza vaccine production capacity. This was in preparation for the Third WHO Consultation on Global Action Plan for Influenza Vaccines (GAP III) taking place in November 2016. These successive programmes have aimed to address the shortage of influenza vaccines for seasonal epidemics and pandemic influenza through encouraging seasonal vaccine uptake; expanding the vaccine production capacity; and promoting further vaccine research and development (R&D). Although it was a bit daunting to be trusted with such a task, I was excited to contribute to a programme that aims to address this critical global public health issue. In addition, I assisted in the scientific and ethical review, and cost analysis of WHO–supported clinical trials of influenza vaccines being developed within low– and middle–income countries. This was a valuable chance to utilise and develop the knowledge and skills I gained though my medical and epidemiological backgrounds in a real world context. I certainly gained a new appreciation for the practicalities and processes in conducting these clinical trials in an ethical and effective manner.

**Figure:**
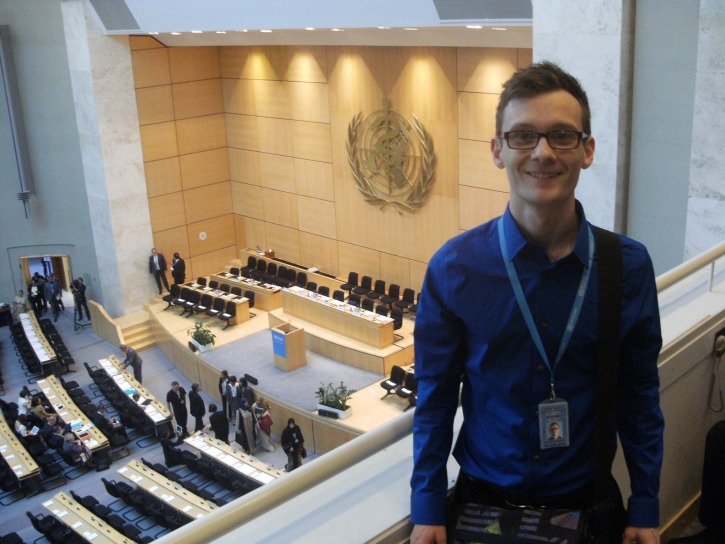
Photo: Kenneth McLean inside the UN

It was a very exciting time during my placement at the PHI unit. My first day was spent observing the Ebola Research and Development Summit where I rapidly gained insight into the many successes, challenges, and lessons learned from the West African Ebola crisis. It immediately helped to highlight how great a privilege my internship was. I was actually at the epicentre of global public health policy development and implementation, and gaining a first–hand understanding of this process. I was also thrilled to be present for the 68th World Health Assembly (WHA), held at the Palais des Nations. This was the first WHA following the catastrophic Ebola outbreak. Therefore, the focus was on the need to build more resilient health systems; improve preparedness and response to emerging outbreaks; and increase R&D on neglected tropical diseases (NTDs). I found it a humbling experience to observe thousands of international delegates and non–governmental organizations (NGO) representatives gathered together in the spirit of collaboration. It was incredible to observe and participate in discussions on some of the most urgent and important health issues facing the world today–the post–2015 sustainable development goals; the health impact of climate change; antimicrobial resistance; pandemic influenza; and equitable access to effective pharmaceuticals.

Alongside the work I undertook, I had the chance to expand my global health horizons though attendance at WHO seminars and workshops, and to be part of the vibrant intern community. While there, I was able to meet with many others who all shared a common aspiration to act to enhance global health. It was fantastic to be able to exchange experiences and perspectives, and to learn from people from an array of professions and places the world over.

As with all good things, my internship sadly had to come to an end. My sincere thanks must go to the Edinburgh University Global Health Society (EUGHS) for their generous financial support, and to my dissertation supervisors – Professor Harry Campbell and Dr Harish Nair – who provided the opportunities and encouragement that enabled me to pursue this internship. Finally, I am immensely grateful to Erin Sparrow and everyone in the Influenza team for such a fantastic chance to work with them, and for the supportive and engaging environment they provided. It was an incredible and unforgettable experience which has only served to further sharpen my interest in global health. It will undoubtedly shape the course of my future medical career.

## MIA COKLJAT: THE HEADQUARTERS OF WORLD HEALTH – WHAT DIFFERENCE CAN A NAÏVE INTERN MAKE?

Summer of 2015 saw my internship at the World Health Organization (WHO) in Geneva. The WHO is divided into headquarters and six regional offices. The headqauarters themselves are then divided into smaller departments that focus on different aspects of health, such as maternal and child health, or non–communicable diseases etc. I was based within the Health Statistics and Information Systems branch of the larger Health Systems and Innovation department.

Whilst there, I was to work on a scoping review, to be published in parallel with the Guidelines for Accurate and Transparent Health Estimates Reporting (GATHER).

It is possible for us to generate health estimates across all countries. Such estimates, termed health metrics, include: mortality, prevalence, and incidence of different diseases or factors that contribute to disease (such as smoking). Together this is termed global burdens of disease (GBD). We can use GBD to create a larger, more global picture what is making people ill, how ill, and where, and how this is changing. Depending on where these burdens of disease are, the greatest allow us allocate resources appropriately; therefore the generation of health metrics can influence health policy. Additionally, they allow us to monitor the impact of worldwide goals, such as the Millennium Development Goals (MDGs).

In order to make these estimates however, we have to use many different data sources. This includes high–quality vital registration data in developed countries, but also low quality self–reported data with significant data gaps in other countries. Moreover, for a given disease for example, there are variations in populations studied, what the outcome measures were, and what methodology was used to collect the data. It is possible to adjust for this by using statistical models; however the more data inputs there are, the more complicated these models become. This can then involve steps such as data cleaning, data pre–processing, data adjustments and weighting of data sources, as well as the mathematical formula itself and the statistical code used. Therefore straight away we can see that unless each step in this complex process is described transparently, there is space for data manipulation, therefore leading to unreplicable health estimates.

Hence, as soon as we are unsure of the source of data, or what all the components of the statistical model are, we begin to be unsure about whether the methodology is transparent. Therefore the health estimate cannot be relied on. Since we use these health estimates to influence our policies and determine our resource allocation, it is of paramount importance that they are replicable . For this reason, we need guidelines that outline a list of basic requirements required in every publication; their inclusion allow us to be sure of the quality and transparency of the health estimate that is reported.

It is possible that all the components outlined on the checklist are already being done, rendering the guidelines surplus to good practice; conversely it is also possible that none of components are being outlined, making the guidelines unrealistic. Therefore my role was to create a scoping review of studies making estimates relating to global burden of disease, and assess them for the current state of reporting.

However, there are wider ideas about global health to learn at the WHO. Within the global health world, there is a relatively new–found favour of community–based interventions. This refers to the idea of using the community as a “setting” and a “resource” for a particular intervention, there to be utilised. This is the idea of taking health care back to local environment of the patient; whether it is management of mental health, or providing rehabilitation after severe acute illness such as cardiac rehabilitation. Thus community–based interventions use a *behavioural change* of the patients themselves to reduce overall population risk of a certain morbidity. There is not necessarily a need to institutionalise patients. The latter takes up vast resources and are not necessarily cost–effect and drastically disrupts the daily lives of patient; moreover it is important to remember that a burden of disease affects the entire community. This is starting to be recognised at a global policy–making level. Unfortunately, doctors are biased to the individual in front of them, and therefore a third party is imperative (such as the WHO) to act as governing body to allocate resources to the community–based interventions, create frameworks describing these interventions, and generate cost–effective policies. The aim of this is to improve the quality of life for the greatest number of people worldwide; this is the utilitarian approach to health care.

**Figure:**
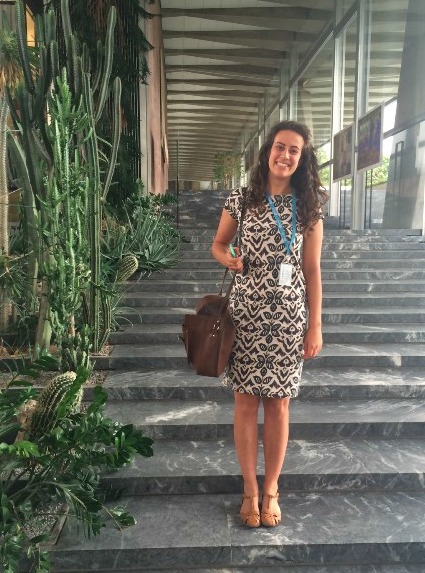
Photo: Mia Cokljat at the WHO

It has been suggested by models of community–based interventions, that the intervention “starts where the people are”. What this means is that rather than creating completely brand new external resources, we see what already exists within the community and modify the already existent practice. Positively, small pilot trials hinted at the optimisation of interventions if they were to be available in one’s own community. This includes perinatal care and injuries as a result of alcohol misuse. However despite this initial promise, there appears to be little progress in creating strong community support system, with only a moderate improvement of health burdens as a result. It would seem the best intentions of the frameworks and guidelines of the WHO, these theoretical ideas do not translate down to feasible actions by health practitioners, and “penetrate” the community. Therefore there are barriers between policies and action; but what these barriers are, we do not know. It could be because there is a lack of education of community–based practitioners on how to technically approach ill health in the community. It could also be, as is the case with mental health, there is that the stigma and lack of awareness of illness means that community interventions are simply not accessible to those that need it. It is also possibly that simply, the interventions are just not be being used for long enough.

Whilst this is an oversimplification of the ideas currently surrounding barriers to implementation of global health interventions at a community–level, it is worth leaving future interns with a thought. It may be that we as interns are too caught up in the large, philosophical questions of global health; instead of asking what needs doing by someone else, we need to ask *how is it* that we can do it. By asking this, we can determine what actions specifically allow us to cross the breach between policy and quantifiable change.

